# Perceived stress and stress responses during COVID-19: The multiple mediating roles of coping style and resilience

**DOI:** 10.1371/journal.pone.0279071

**Published:** 2022-12-15

**Authors:** Qi Gao, Huijing Xu, Cheng Zhang, Dandan Huang, Tao Zhang, Taosheng Liu

**Affiliations:** 1 Department of Psychiatry, Faculty of Psychology, Naval Medical University, Shanghai, China; 2 Department of Political Theory, Qingdao Branch of Naval Aeronautical University, Qingdao, China; 3 Department of Medical Psychology, Changzheng Hospital, Naval Medical University, Shanghai, China; University of Catania Libraries and Documentation Centre: Universita degli Studi di Catania, ITALY

## Abstract

Although many studies have examined the effects of perceived stress on some specific stress responses during the COVID-19, a comprehensive study is still lacking. And the co-mediating role of coping style and resilience as important mediators of stress processes is also unclear. This study aimed to explore the effects of perceived stress on emotional, physical, and behavioral stress responses and the mediating roles of coping style and resilience in Chinese population during the recurrent outbreak of COVID-19 from a comprehensive perspective. 1087 participants were recruited to complete the anonymous online survey including the Perceived Stress Scale, the Stress Response Questionnaire, the Simplified Coping Style Questionnaire and the Emotional Resilience Questionnaire. Pearson’s correlation and Hayes PROCESS macro 3.5 model 6 were used in the mediating effect analysis. Results showed that positive coping style and resilience both buffered the negative effects of perceived stress on emotional, physical, and behavioral responses through direct or indirect pathways, and resilience had the strongest mediating effects. The findings urged relevant authorities and individuals to take measures to promote positive coping style and resilience to combat the ongoing pandemic stress and protect public physical and mental health.

## 1. Introduction

The recurring COVID-19 pandemic now constitutes a public health emergency of international concern despite being in its third year [1]. The outbreak and persistence of COVID-19 has brought a great burden to the public through the risk of infection, social isolation, economic downturn, and other negative events that put the public under great psychological stress [2–4]. Such chronic stress exposure can lead to various stress responses in individuals. Many studies confirmed that perceived stress [the evaluation of one’s perceived level of stress] during the pandemic was positively relevant to individual emotional responses such as anxiety and depression [5, 6], and physical responses such as insomnia [7], especially for those with psychic fragility [8]. According to the System-based Model of Stress proposed by Jiang [9], stress responses consist of emotional, physical and behavioral changes that people exhibit as a result of stress. However, no studies have yet explored the effects of perceived stress on different stress responses in the same group during the pandemic. Given the multidimensional nature of stress responses, a comprehensive study could provide a more systematic and holistic understanding of individual stress in the context of the ongoing COVID-19 pandemic.

Individuals may exhibit different levels of perceived stress and stress responses even when confronted with the same stressors [10]. The Stress, Emotions, and Performance meta-model states that the stress process begins with the perception of stress, mediated by various levels of cognitive appraisal and coping resources, and then leads to positive or negative stress responses [11]. In this process, some protective or risk factors mediate the effects of perceived stress on stress responses. One of them is coping style. Coping style refers to an individual’s cognitive or behavioral pattern in the face of frustration or stressors which can moderate the stages of stress as an individual trait [12]. Yan et al. [13] investigated the effect of coping style on the relationship between perceived stress and psychological distress and found that positive coping style alleviated individuals’ emotional distress, while negative coping did the opposite. The results was consistent with most related-topic researches, with positive or adaptive coping being associated with lower levels of anxiety and depression, and negative or maladaptive coping exacerbating psychological distress of people [14–16]. Additionally, a review of stress-related mood disorders suggested that differences in coping styles directly leaded to differences in individual physiological responses to stressors, which in turn affected individual susceptibility to illness [17]. However, the role of coping styles in the relationship between perceived stress and physical or behavioral responses during the pandemic was still unclear.

Resilience is recognized as a pervasive individual characteristic that helps individual adapt to or overcome adversity, stress, trauma, and recover from these negative experiences [18, 19]. Resilience has played an important role in reducing individual mental illness and protecting public well-being during COVID-19 [20–23]. Wilks and Croom [24] discovered that individuals with higher perceived stress may develop lower levels of resilience [25], which in turn resulted in higher psychological problems [26]. But similarly, there have been few studies on the role of resilience in mitigating the negative effects of perceived stress on physical and behavioral responses. As one of the important protective factors for stress, it is reasonable to speculate that resilience may also mitigate physical and behavioral stress responses of individuals during the pandemic.

Coping style and resilience are considered to be closely related concepts [27], which can jointly relieve individual stress responses. Campbell-Sills et al. [12] reported that coping style was significantly associated with resilience. Positive and adaptive coping style contributed to the development of resilience, while negative maladaptive coping style was an important risk factor for low levels of resilience [28]. A review from Shing et al. [29] also concluded that resilience during and after disasters may partly depend on individual coping style. Such ability to cope with stress was critical for people to recover and adapt after crises, which could be significantly strengthened by positive coping style [30, 31]. To our knowledge, no researches have studied how coping style and resilience together function in the effects of perceived stress on stress responses.

**Fig 1:**
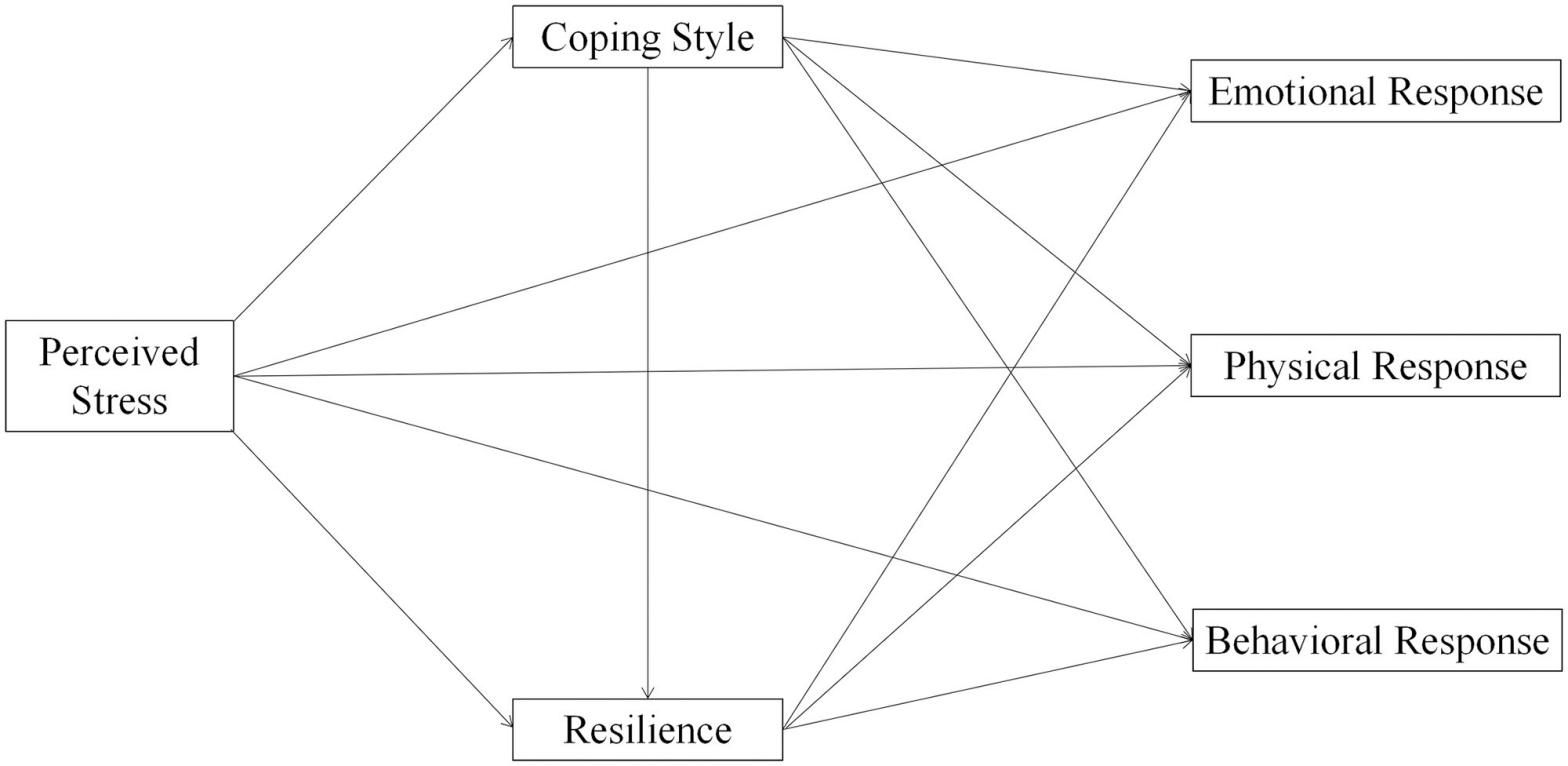
Proposed chain mediation model.

In summary, this study aims to explore the effects of perceived stress on emotional, physical, and behavioral stress responses and the mediating roles of coping style and resilience in the Chinese population during the third year of COVID-19, in order to provide scientific suggestions for relevant organizations and individuals to take more effective measures to reduce stress responses and protect physical and mental health in the context of the ongoing pandemic. The proposed models of this study is shown in Fig 1.

## 2. Materials and methods

### 2.1 Participants

Convenience sampling and snowball sampling were used in this study. A total of 1087 subjects were recruited to voluntarily participate in an anonymous questionnaire through the Questionnaire Star platform (https://www.wjx.cn/vm/YLUDsAO.aspx) from March 2022 to April 2022. The inclusion criteria included age above 18 years old, currently in mainland China, and voluntary investigation participation. The exclusion criteria included participants’ ages of less than 18 or more than 70, participants response times of less than 200 s or more than 1800 s, repetitive answers, and confusing logic. Finally, 873 valid respondents were included in the analysis (valid ratio = 80.31%, mean age = 38.22 ± 11.95), from 89 cities in 28 provinces of China.

This study was approved by the Ethics Committee of Naval Medical University (NMUMEREC-2021-043). The ethical principles of the Declaration of Helsinki were followed in the course of the study. All participants were asked to complete an informed consent notification prior to the questionnaire. Furthermore, participants were guaranteed the voluntary and confidential of their responses and their rights to quit the survey at any time.

### 2.2 Measures

#### 2.2.1 Demographics

Demographic variables included gender (Male, Female), age (18–35, 36–50, >50), occupations (Occupations with COVID-19 exposure risk: healthcare works, administrators whose work is directly involved with the pandemic, pandemic volunteers, etc; Occupations without COVID-19 exposure risk: enterprise worker, teachers, students, etc), quarantine or not (Being quarantined, Not being quarantined), and financial worries (Extreme worry, Serious worry, Moderate worry, Mild worry, Not at all.)

#### 2.2.2 Perceived stress

The Perceived Stress Scale (PSS-10) was applied to measure to which degree people felt their lives as stressful in the past month [32]. The scale consists of 10 items ranged from 0 (never) to 4 (always). The total scores are calculated by the sum of the 10 items ranging from 0 to 40. Higher scores denote higher perceived stress. In this study, the Cronbach’s α was 0.834.

#### 2.2.3 Stress responses

The 28-item Stress Response Questionnaire [9] was used to assess the degree of individual’s stress responses over the last month. The scale includes three subscales: Emotional Response (ER: anxiety, depression, anger, etc. i.e.,”Feeling sullen and depressed.”), Physical Response (PR: dizziness, body pain, fatigue and lassitude, etc. i.e., “Feeling weak and tired easily.“), and Behavioral Response (BR: avoidance, reduced physical activity, etc. i.e., “Too lazy to move.“). Each item is scored on a 5-point Likert scale from 1 (surely yes) to 5 (surely not). The total scores for each subscale are summed by the corresponding items. Only three subscales were used in the present study and the Cronbach’s α for ER, PR and BR was 0.946, 0.915 and 0.847, respectively.

#### 2.2.4 Coping style

The Simplified Coping Style Questionnaire (SCSQ) was employed to measure the coping style (CS) that people were accustomed to using in their lives [33]. There are two dimensions of this scale: positive coping style with 12 items and negative coping style with 8 items. Items are rated on 4-point Likert scales from 0 (never) to 3 (always). In this study, the participants’ final coping style scores were equal to the positive coping scores minus the negative coping scores. The higher the final score, the more inclined the individual was to the positive coping style, and the less inclined the individual was to the negative coping style [15]. The Cronbach’s α was 0.876 for the current study.

#### 2.2.5 Resilience

The Emotional Resilience Questionnaire (ERQ) was selected to measure resilience (R). This scale was firstly designed by Zhang and Lu [34] based on Chinese local culture to assess the resilience of adolescents, the validity has also been demonstrated in adults [35]. The questionnaire includes 11 items rated from 0 (never) to 6 (always). The total scores are the sum of the 11 items with higher scores indicating greater resilience. The Cronbach’s α was 0.845 in this study.

### 2.3 Data analysis

All data were analyzed by SPSS21.0 (IBM SPSS Statistics for Windows, Version 21.0. Armonk, NY: IBM Corp). Descriptive statistics were conducted to describe demographic characteristics. Pearson’s correlation analyses were used to examine the correlation between variables of interest. Model 6 of PROCESS v 3.5 was selected to test the mediating effects of coping style and resilience [36], and the significance of indirect effects were examined by bootstrap method (5000 bootstrap samples) with 95% confidence intervals (CIs). Demographic characteristics were included in all mediation models as covariates. All variables were standardized before analysis. All statistical tests were two-tailed with *p* < 0.05 as statistically significant.

## 3. Results

### 3.1 Sample characteristics

The sample characteristics are presented in Table 1. More than two-third of the participants were female (63.80%). 48.45% of the samples were aged between 18–35, and 35.51% were 36–50. 24.74% were occupationally at the risk of COVID-19 exposure. About half of the participants were being quarantined at the time of our investigation. 6.99% of the individuals reported extremely worried about self finances and 20.39% didn’t worried at all.

**Table 1:** Demographic and variables characteristics (*n* = 873).

	*n*	Ratio(%)
**Gender**		
Male	316	36.20
Female	557	63.80
**Age**		
18–35	423	48.45
36–50	310	35.51
>50	140	16.04
**Occupations**		
Occupations with COVID-19 exposure risk	216	24.74
Occupations without COVID-19 exposure risk	657	75.26
**Quanantine or not**		
Being quarantined	386	44.22
Not being quarantined	487	55.78
**Financial worries**		
Extreme worry	61	6.99
Serious worry	163	18.67
Moderate worry	324	37.11
Mild worry	147	16.84
Not at all	178	20.39

### 3.2 Correlation analysis

Table 2 shows the results of Person correlation analysis between variables. Perceives stress, emotional response, physical response, and behavioral response were positively inter-correlated with each other, and were all negatively associated with coping style and resilience (all *p* < 0.01). Coping style was positively related to resilience (*p* < 0.01).

**Table 2:** Correlation analysis between variables.

	***M***±***SD***	**Perceived stress**	**Emotional response**	**Physical response**	**Behavioral response**	**Coping style**	**Resilience**
**Perceived stress**	14.18±6.71	1					
**Emotional response**	19.91±9.74	0.72**	1				
**Physical response**	15.13±6.90	0.69**	0.88**	1			
**Behavioral response**	11.41±5.03	0.62**	0.84**	0.82**	1		
**Coping style**	13.17±7.71	-0.48**	-0.41**	-0.36**	-0.41**	1	
**Resilience**	36.23±8.95	-0.57**	-0.53**	-0.51**	-0.47**	0.49**	1

** *p*<0.01.

### 3.3 Multiple mediation analysis

**Fig 2:**
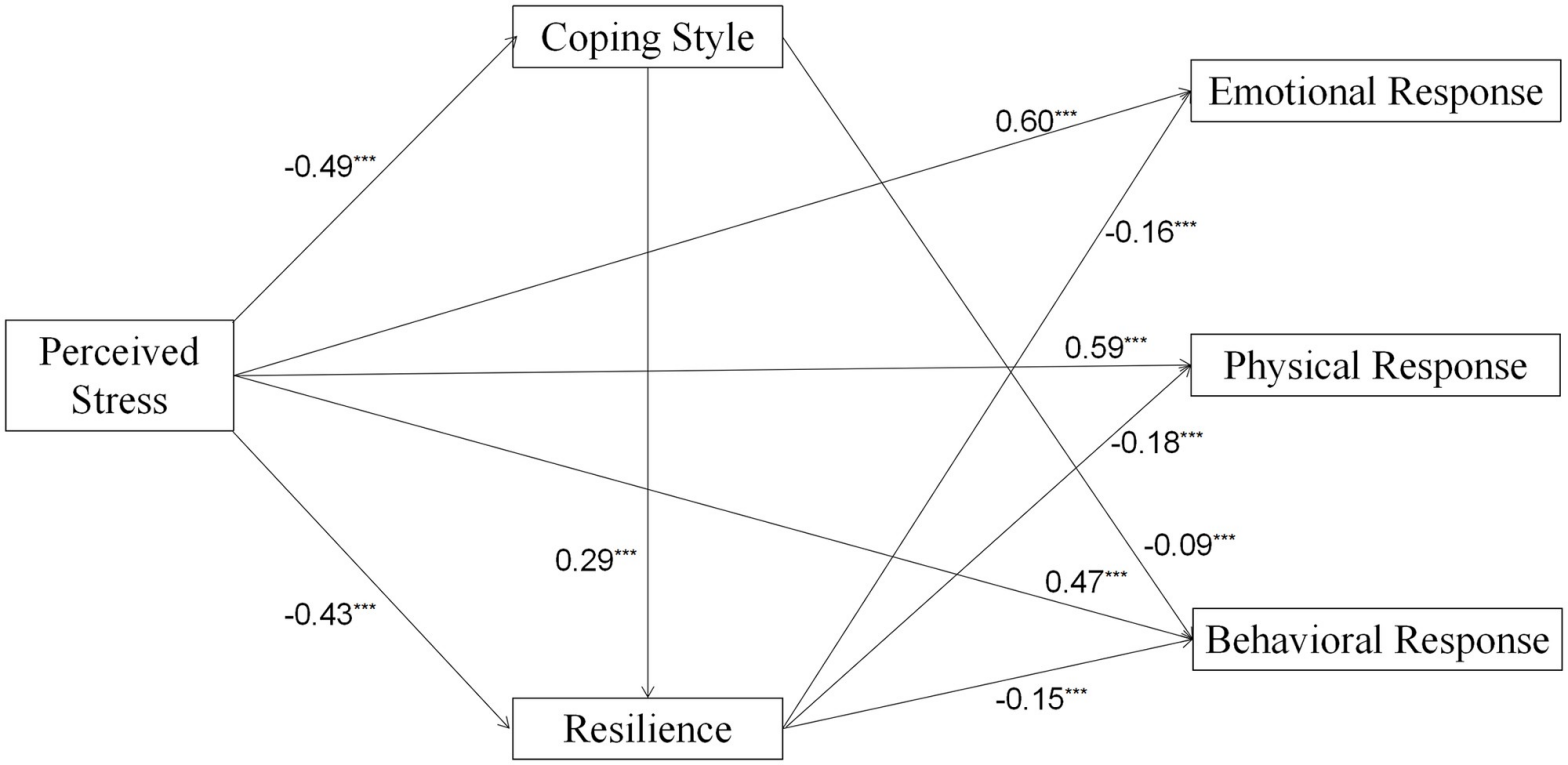
Chain mediation model of perceived stress on emotional response, physical response, and behavioral response through coping style and resilience.

The results of the multiple mediation analysis are shown in Table 3 and Fig 2. After controlling for gender, age, occupations, quarantine or not, and financial worries, PSS showed negative direct effects on CS (*β* = -0.49, *p* < 0.001) and R (*β* = -0.43, *p* < 0.001), as well as positive direct effects on ER (*β* = 0.60, *p* < 0.001), PR (*β* = 0.59, *p* < 0.001), and BR (*β* = 0.47, *p* < 0.001). CS was positively related to R (*β* = 0.29, *p* < 0.001) and negatively related to BR (*β* = -0.09, *p* < 0.001). R was negatively associated with ER (*β* = -0.16, *p* < 0.001), PR (*β* = -0.18, *p* < 0.001), and BR (*β* = -0.15, *p* < 0.001). Other direct paths were not significant.

**Table 3:** Total and direct paths analysis results.

	*β*	SE	*t*	*p*	95%CI
**Total effects**					
PSS-ER	0.70	0.04	26.72	<0.001	0.95, 1.10
PSS-PR	0.70	0.03	25.04	<0.001	0.66, 0.77
PSS-BR	0.60	0.02	20.15	<0.001	0.41, 0.49
**Direct effects**					
PSS-CS	-0.49	0.04	-14.77	<0.001	-0.64, -0.49
PSS-R	-0.43	0.04	-12.99	<0.001	-0.67, -0.49
CS-R	0.29	0.04	9.41	<0.001	0.26, 0.40
PSS-ER	0.60	0.05	18.90	<0.001	0.78, 0.96
CS-ER	-0.03	0.03	-1.05	0.29	-0.11, 0.03
R-ER	-0.16	0.03	-5.49	<0.001	-0.24, -0.11
PSS-PR	0.59	0.03	17.85	<0.001	0.54, 0.68
CS-PR	0.01	0.03	0.36	0.72	-0.04, 0.06
R-PR	-0.18	0.02	-5.95	<0.001	-0.19, -0.10
PSS-BR	0.47	0.03	13.25	<0.001	0.30, 0.41
CS-BR	-0.09	0.02	-2.91	<0.001	-0.10, -0.02
R-BR	-0.15	0.02	-4.56	<0.001	-0.12, -0.05

PSS: Perceived stress; ER: Emotional response; PR: Physical response; BR: Behavioral response; CS: Coping style; R: Resilience.

The bootstrap method mediating analysis (Table 4) showed that the indirect effects of PSS on ER through R (*Effect* = 0.07, *SE* = 0.01, 95%CI [0.04, 0.10]) and chain path of CS and R (*Effect* = 0.02, SE = 0.01, 95%CI [0.01, 0.03]) were significant. The indirect effects of PSS on PR via R (*Effect* = 0.08, *SE* = 0.02, 95%CI [0.05, 0.11]) and chain path of CS and R (*Effect* = 0.03, *SE* = 0.01, 95%CI [0.01, 0.04]) were significant. The indirect effects of PSS on BR through CS (*Effect* = 0.04, *SE* = 0.02, 95%CI [0.01, 0.08]), R (*Effect* = 0.07, *SE* = 0.02, 95%CI [0.03, 0.10]) and chain path of CS and R (*Effect* = 0.02, *SE* = 0.01, 95%CI [0.01, 0.03]) were significant. Other indirect paths were not significant.

**Table 4:** Indirect analysis results.

Bootstrapped indirect effects	*Effect*	BootSE	95%CI	Mediating effect(%)
PSS-CS-ER	0.01	0.01	-0.01, 0.04	
PSS-R-ER	0.07	0.01	0.04, 0.10	10
PSS-CS-R-ER	0.02	0.01	0.01, 0.03	2.86
PSS-CS-PR	-0.01	0.02	-0.03, 0.02	
PSS-R-PR	0.08	0.02	0.05, 0.11	11.43
PSS-CS-R-PR	0.03	0.01	0.01, 0.04	4.29
PSS-CS-BR	0.04	0.02	0.01, 0.08	6.67
PSS-R-BR	0.07	0.02	0.03, 0.10	11.67
PSS-CS-R-BR	0.02	0.01	0.01, 0.03	3.33

PSS: Perceived stress; ER: Emotional response; PR: Physical response; BR: Behavioral response; CS: Coping style; R: Resilience.

## 4. Discussion

COVID-19 posed a widespread and extensive threat to the public as a worldwide health emergency. The persistence of such a negative stressor would trigger a range of psychological, physiological and behavioral changes of people [37]. This study provided a comprehensive perspective about the effects of perceived stress on emotional, physical, and behavioral stress responses, as well as the separate mediating and co-mediating effects of coping style and resilience. As expected, perceived stress was positively associated with emotional, physical, and behavioral responses. People with higher perceived stress reported more negative emotions, more severe physical symptoms, and more maladaptive behaviors, which was in line with most of the previous studies [38–41].

In the present study, coping style alone mediated the association between perceived stress and behavioral responses, and positive coping could buffer the negative effects of perceived stress on behavioral responses. However, coping style did not mediate the effects of perceived stress on emotional or physical responses, which was inconsistent with previous studies. A majority of studies regarded coping style as an important stress mediator between stressors and all kinds of stress responses [42, 43]. People who prefer positive coping style tend to face problems directly and take ways to solve it positively, while people with negative coping style are inclined to avoid problems by denying and withdrawing, which in turn leads to worsening of emotional responses such as anxiety and depression, and an increase in physical responses such as lowered immunity [13, 17, 44]. Here are three possible explanations for the inconsistency. One is that the method of calculating coping style in our study may weaken the effects of different coping styles, since positive and negative coping styles have opposite effects. Another is that coping styles themselves are behavioral expressions and thus have stronger influence on behavioral response. The third explanation is that the role of coping style in the effects of perceived stress on emotional and physical responses are complicated. An mediation research conducted in older people reported that neither positive nor negative coping was correlated with anxiety response [45]. Lau et al. [14] also discovered that coping style did not have a direct effect on anxiety, but negative coping style showed a positive and significant effect on anxiety after positive coping style was added as a moderating variable to the structural equation. Therefore, the function of coping style in stress process still needs further research.

The results demonstrated the mediating role of resilience in the relationship between perceived stress and stress responses. Consistent with previous research, higher perceived stress was associated with lower levels of resilience, as the ongoing stress from the epidemic increased the burden on people’s coping resources [46]. And higher resilience was associated with less stress responses [26, 47, 48]. More importantly, our study confirms the role of resilience in buffering physical and behavioral responses in addition to emotional responses. As an effective protective factor, the higher the level of individual resilience, the better the individual’s ability to counteract perceived stress and the less emotional, physical, and behavioral stress responses are exhibited. According to the Stress Inoculation theory, moderate stress exposure contributes to resilience development. Some longitudinal studies also found that the psychological problems of individuals increased sharply in the early stages of the pandemic, then decreased rapidly, which was interpreted as the stimulation and validation of resilience [49, 50]. However, longer term longitudinal studies are needed to clarify whether individual coping resources and resilience will be depleted as the pandemic continues. In any case, at least in the third year of the COVID-19 pandemic, resilience still plays an important role in mitigating various stress responses of people.

Coping style and resilience showed chain mediating effects between perceived stress and emotional, physical, behavioral responses in the current study. Many studies endorsed that people with fewer psychological problems in the pandemic had more successful coping and higher levels of resilience [51, 52]. As a resilience protective factor, positive coping style was proved to contribute to resilience [12, 28, 30]. Except for behavioral response, coping style in this study cannot directly affect the individual’s emotional and physical responses, but need to be through resilience, and the more positive the coping tendency, the higher the resilience and the less the individual’s emotional, physical, and behavioral responses. As for the mediating effect size, the mediating effect of resilience was the strongest among all indirect pathways, suggesting that resilience performed a stronger role in buffering the effects of perceived stress on stress responses. In contrast, the simple mediation of coping style and chain mediation effects of coping style and resilience were weaker. Nevertheless, our study establishes the important roles and pathways of coping style and resilience in the relationship between perceived stress and different stress responses. The results suggest that fostering positive coping style and promoting resilience are necessary for individuals to better adapt to pandemic-related stressors, reduce stress responses, and recover quickly from pandemic trauma. Individuals are encouraged to proactively develop available protective measures, such as learning stress management methods [36], doing physical exercise or maintaining healthy lifestyle [53], and cultivating meaningful relationships for social support [54, 55]. Governments and mental health organizations already have some useful measures, such as encouraging social connections [56], organizing psychological support groups, and providing services for mindfulness practice [57]. Some coping-focused efforts like coping skills training courses and online virtual stress adaptation training may also be helpful as our results have verified the positive effect of coping style on resilience [58, 59].

## 5. Limitation

Some limitations should be considered when interpreting the results of this study. Firstly, the convenience and snowball sampling used in the study may affect the generalization of the results and the representativeness of the sample, even though our sample was distributed across the majority of Chinese provinces. And as a cross-sectional study, no causal conclusions can be inferred. Secondly, several demographic variables (such as gender, age, and economy) were selected as covariates to increase the reliability of the results, but the factors influencing the dependent variables are actually numerous and complex. Except for demographic influences, social or political factors may also affect the perceived stress and stress responses of people. Thirdly, this study focused only on the mediating role of coping style and did not explore the effects of specific coping strategies. Studies have shown that positive or negative coping strategies did not necessarily alleviate or exacerbate stress responses. For example, Panourgia et al. [60] discovered that avoidance behavior can be an effective adaptive strategy in some situations, allowing people to deal with problems more quickly. Considering the complexity of the effects of coping, the ways in which coping and specific coping strategies function deserve further study. Fourthly, the present study explored emotional, physical, and behavioral stress responses in general but not in depth, and future studies could examine each stress response type in more detail. Last but not least, this study included the variable of “quanantine or not” as a covariate, but some studies showed that quarantined people experienced higher levels of stress [61, 62], which means that some quarantined participants in this study may have reported higher levels of perceived stress and stress responses, so the findings may be biased and should be interpreted with caution. Further studies could continue to explore perceived stress and stress response of individuals in different quarantine states, as well as the influencing factors.

## 6. Conclusions

This study is the first to examine overall the effects of perceived stress on emotional, physical, and behavioral responses and the mediating roles of coping style and resilience during the recurrent outbreak of the COVID-19. The results indicated that more positive coping style and especially higher levels of resilience buffered the negative effects of perceived stress on different stress responses, suggesting that relevant authorities and individuals should take measures to foster positive coping and promote the development of resilience to against the ongoing pandemic stress and to protect individuals’ physical and mental health.

## Supporting information

S1 Dataset (XLSX)

## References

[1] World Health Organization. Statement on the twelfth meeting of the International Health Regulations (2005) Emergency Committee regarding the coronavirus disease (COVID-19) pandemic. (2022) https://www.who.int/news/item/12-07-2022-statement-on-the-twelfth-meeting-of-the-international-health-regulations-(2005)-emergency-committee-regarding-the-coronavirus-disease-(covid-19)-pandemic. (accessed on 24 July 2022).

[2] Chaudhry AW, Kazmi B, Sharjeel S, Akhtar Z, Shahid S. Learning from the Past: A Systematic Review on Risk and Protective Factors for Psychological Distress in Past Infectious Epidemics and COVID-19. J Res Psychol. 2021; 3:1–54. doi: 10.31580/jrp.v3i1.1805

[3] Hagger MS, Keech JJ, Hamilton K. Managing stress during the coronavirus disease 2019 pandemic and beyond: Reappraisal and mindset approaches. Stress Health. 2020; 36:396–401. doi: 10.1002/smi.2969 32588961 PMC7361383

[4] Brooks SK, Webster RK, Smith LE, Woodland L, Wessely S, Greenberg N, et al. The psychological impact of quarantine and how to reduce it: rapid review of the evidence. Lancet. 2020; 395:912–20. doi: 10.1016/S0140-6736(20)30460-8 32112714 PMC7158942

[5] Saravanan C, Wilks R. Medical students’ experience of and reaction to stress: the role of depression and anxiety. ScientificWorldJournal. 2014; 2014:737382. doi: 10.1155/2014/737382 24688425 PMC3929074

[6] Xu C, Xu Y, Xu S, Zhang Q, Liu X, Shao Y, et al. Cognitive Reappraisal and the Association Between Perceived Stress and Anxiety Symptoms in COVID-19 Isolated People. Front Psychiatry. 2020; 11:858. doi: 10.3389/fpsyt.2020.00858 32982809 PMC7492600

[7] Zhao X, Lan M, Li H, Yang J. Perceived stress and sleep quality among the non-diseased general public in China during the 2019 coronavirus disease: a moderated mediation model. Sleep Med. 2021; 77:339–45. doi: 10.1016/j.sleep.2020.05.021 32482485 PMC7240276

[8] Natale A, Concerto C, Rodolico A, Birgillito A, Bonelli M, Martinez M, et al. Risk Perception among Psychiatric Patients during the COVID-19 Pandemic. Int J Environ Res Public Health. 2022 Feb 24;19(5):2620. doi: 10.3390/ijerph19052620 . 35270313 PMC8909657

[9] Jiang Q. Medical Psychology Theories,Methods and Clinic. 2st ed. Beijing: People’s Medical Publishing House; 2012.

[10] Lazarus RS. Psychological Stress and Coping in Adaptation and Illness. The International Journal of Psychiatry in Medicine. 1974; 5: 321–333. doi: 10.2190/T43T-84P3-QDUR-7RTP 4618837

[11] Fletcher D, Sarkar M. Psychological resilience: A review and critique of definitions, concepts, and theory. European Psychologist. 2013; 18:12–23. doi: 10.1027/1016-9040/A000124

[12] Campbell-Sills L, Cohan SL, Stein MB. Relationship of resilience to personality, coping, and psychiatric symptoms in young adults. Behav Res Ther. 2006; 44:585–99. doi: 10.1016/j.brat.2005.05.001 15998508

[13] Yan L, Gan Y, Ding X, Wu J, Duan H. The relationship between perceived stress and emotional distress during the COVID-19 outbreak: Effects of boredom proneness and coping style. J Anxiety Disord. 2021; 77:102328. doi: 10.1016/j.janxdis.2020.102328 33160275 PMC7598556

[14] Lau Y, Wang Y, Kwong DH, Wang Y. Testing direct and moderating effects of coping styles on the relationship between perceived stress and antenatal anxiety symptoms. J Psychosom Obstet Gynaecol. 2015; 36:29–35. doi: 10.3109/0167482X.2014.992410 25541216

[15] Li Z, Yi X, Zhong M, Li Z, Xiang W, Wu S, et al. Psychological Distress, Social Support, Coping Style, and Perceived Stress Among Medical Staff and Medical Students in the Early Stages of the COVID-19 Epidemic in China. Front Psychiatry. 2021; 12:664808. doi: 10.3389/fpsyt.2021.664808 34140903 PMC8203804

[16] Wang Y, Wang P. Perceived stress and psychological distress among chinese physicians: The mediating role of coping style. Medicine (Baltimore). 2019; 98:e15950. doi: 10.1097/MD.0000000000015950 31169719 PMC6571215

[17] Kalinichenko LS, Kornhuber J, Müller CP. Individual differences in inflammatory and oxidative mechanisms of stress-related mood disorders. Front Neuroendocrinol. 2019; 55:100783. doi: 10.1016/j.yfrne.2019.100783 31415777

[18] Connor KM, Davidson JR. Development of a new resilience scale: the Connor-Davidson Resilience Scale (CD-RISC). Depress Anxiety. 2003; 18:76–82. doi: 10.1002/da.10113 12964174

[19] Southwick SM, Bonanno GA, Masten AS, Panter-Brick C, Yehuda R. Resilience definitions, theory, and challenges: interdisciplinary perspectives. Eur J Psychotraumatol. 2014; 5. doi: 10.3402/ejpt.v5.25338 25317257 PMC4185134

[20] Killgore W, Taylor EC, Cloonan SA, Dailey NS. Psychological resilience during the COVID-19 lockdown. Psychiatry Res. 2020; 291:113216. doi: 10.1016/j.psychres.2020.113216 32544705 PMC7280133

[21] Liu CH, Zhang E, Wong G, Hyun S, Hahm HC. Factors associated with depression, anxiety, and PTSD symptomatology during the COVID-19 pandemic: Clinical implications for U.S. young adult mental health. Psychiatry Res. 2020; 290:113172. doi: 10.1016/j.psychres.2020.113172 32512357 PMC7263263

[22] Manchia M, Gathier AW, Yapici-Eser H, Schmidt MV, de Quervain D, van Amelsvoort T, et al. The impact of the prolonged COVID-19 pandemic on stress resilience and mental health: A critical review across waves. Eur Neuropsychopharmacol. 2022; 55:22–83. doi: 10.1016/j.euroneuro.2021.10.864 34818601 PMC8554139

[23] Osimo SA, Aiello M, Gentili C, Ionta S, Cecchetto C. The Influence of Personality, Resilience, and Alexithymia on Mental Health During COVID-19 Pandemic. Front Psychol. 2021; 12:630751. doi: 10.3389/fpsyg.2021.630751 33716896 PMC7943855

[24] Wilks SE, Croom B. Perceived stress and resilience in Alzheimer’s disease caregivers: testing moderation and mediation models of social support. Aging Ment Health. 2008; 12:357–65. doi: 10.1080/13607860801933323 18728949

[25] Morales-Rodríguez FM, Martínez-Ramón JP, Méndez I, Ruiz-Esteban C. Stress, Coping, and Resilience Before and After COVID-19: A Predictive Model Based on Artificial Intelligence in the University Environment. Front Psychol. 2021; 12:647964. doi: 10.3389/fpsyg.2021.647964 34017287 PMC8129547

[26] Havnen A, Anyan F, Hjemdal O, Solem S, Gurigard Riksfjord M, Hagen K. Resilience Moderates Negative Outcome from Stress during the COVID-19 Pandemic: A Moderated-Mediation Approach. Int J Environ Res Public Health. 2020; 17. doi: 10.3390/ijerph17186461 32899835 PMC7558712

[27] Folkman S, Moskowitz JT. Coping: pitfalls and promise. Annu Rev Psychol. 2004; 55:745–74. doi: 10.1146/annurev.psych.55.090902.141456 14744233

[28] Sampogna G, Del Vecchio V, Giallonardo V, Luciano M, Albert U, Carmassi C, et al. What Is the Role of Resilience and Coping Strategies on the Mental Health of the General Population during the COVID-19 Pandemic? Results from the Italian Multicentric COMET Study. Brain Sci. 2021; 11. doi: 10.3390/brainsci11091231 34573251 PMC8466446

[29] Shing EZ, Jayawickreme E, Waugh CE. Contextual Positive Coping as a Factor Contributing to Resilience After Disasters. J Clin Psychol. 2016; 72:1287–306. doi: 10.1002/jclp.22327 27410521

[30] Brooks S, Amlôt R, Rubin GJ, Greenberg N. Psychological resilience and post-traumatic growth in disaster-exposed organisations: overview of the literature. BMJ Mil Health. 2020; 166:52–6. doi: 10.1136/jramc-2017-000876 29420257

[31] Tugade MM, Fredrickson BL, Barrett LF. Psychological resilience and positive emotional granularity: examining the benefits of positive emotions on coping and health. J Pers. 2004; 72:1161–90. doi: 10.1111/j.1467-6494.2004.00294.x 15509280 PMC1201429

[32] Wang Z, Wang Y, Wu ZG, Chen DD, Chen Y, Xiao ZP. Reliability and validity of the Chinese version of Perceived Stress Scale. Journal of Shanghai Jiao Tong University Medical Science. 2015; 35:1448. doi: 10.3969/j.issn.1674-8115.2015.10.004

[33] Xie YN. A preliminary study of the reliability and validity of the simplified coping style questionnaire (in Chinese). Chin J Clin Psychol. 1998; 6:114–5.

[34] Zhang M, Lu JM. The development of adolescents’ emotional resilience questionnaire. Psychological Science. 2010; 33:24–27.

[35] Zhang, Y. Attentional bias and autonomic response of emotional resilience in college students. M.Ed. Thesis, Tianjin Normal University. 2013. https://kns.cnki.net/kcms/detail/detail.aspx?dbcode=CMFD&dbname=CMFD201402&filename=1014117971.nh&uniplatform=NZKPT&v=oY81E8UTFUfo7ksR3xl5usKuvM8Y8lzRKmUVqrHrJp0kKFBLqOWLsALX7wV_Zzj0

[36] Hayes AF. Introduction to Mediation, Moderation, and Conditional Process Analysis: A Regression-Based Approach. NY:Guilford Press; 2013.

[37] Blanc J, Briggs AQ, Seixas AA, Reid M, Jean-Louis G, Pandi-Perumal SR. Addressing psychological resilience during the coronavirus disease 2019 pandemic: a rapid review. Curr Opin Psychiatry. 2021; 34:29–35. doi: 10.1097/YCO.0000000000000665 33230041 PMC7751836

[38] Horesh D, Brown AD. Traumatic stress in the age of COVID-19: A call to close critical gaps and adapt to new realities. Psychol Trauma. 2020; 12:331–335. doi: 10.1037/tra0000592 32271070

[39] Si G, Xu Y, Li M, Zhang Y, Peng S, Tan X. Sleep quality and associated factors during the COVID-19 epidemic among community non-medical anti-epidemic Workers of Wuhan, China. BMC Public Health. 2021; 21:1270. doi: 10.1186/s12889-021-11312-8 34193093 PMC8242282

[40] Varma P, Junge M, Meaklim H, Jackson ML. Younger people are more vulnerable to stress, anxiety and depression during COVID-19 pandemic: A global cross-sectional survey. Prog Neuropsychopharmacol Biol Psychiatry. 2021; 109:110236. doi: 10.1016/j.pnpbp.2020.110236 33373680 PMC7834119

[41] Znazen H, Slimani M, Bragazzi NL, Tod D. The Relationship between Cognitive Function, Lifestyle Behaviours and Perception of Stress during the COVID-19 Induced Confinement: Insights from Correlational and Mediation Analyses. Int J Environ Res Public Health. 2021; 18. doi: 10.3390/ijerph18063194 33808777 PMC8003540

[42] Cai Z, Zheng S, Huang Y, Zhang X, Qiu Z, Huang A, et al. Emotional and Cognitive Responses and Behavioral Coping of Chinese Medical Workers and General Population during the Pandemic of COVID-19. Int J Environ Res Public Health. 2020; 17. doi: 10.3390/ijerph17176198 32859064 PMC7504432

[43] Folkman S, Lazarus RS, Gruen RJ, DeLongis A. Appraisal, coping, health status, and psychological symptoms. J Pers Soc Psychol. 1986; 50:571–9. doi: 10.1037//0022-3514.50.3.571 3701593

[44] Liu Y, Hou T, Gu H, Wen J, Shao X, Xie Y, et al. Resilience and Anxiety Among Healthcare Workers During the Spread of the SARS-CoV-2 Delta Variant: A Moderated Mediation Model. Front Psychiatry. 2022; 13:804538. doi: 10.3389/fpsyt.2022.804538 35250664 PMC8889094

[45] Zapater-Fajarí M, Crespo-Sanmiguel I, Pulopulos MM, Hidalgo V, Salvador A. Resilience and Psychobiological Response to Stress in Older People: The Mediating Role of Coping Strategies. Front Aging Neurosci. 2021; 13:632141. doi: 10.3389/fnagi.2021.632141 33692681 PMC7937969

[46] Chen Y, Xu H, Liu C, Zhang J, Guo C. Association Between Future Orientation and Anxiety in University Students During COVID-19 Outbreak: The Chain Mediating Role of Optimization in Primary-Secondary Control and Resilience. Front Psychiatry. 2021; 12:699388. doi: 10.3389/fpsyt.2021.699388 34421680 PMC8373437

[47] Megías-Robles A, Gutiérrez-Cobo MJ, Cabello R, Gómez-Leal R, Fernández-Berrocal P. A longitudinal study of the influence of concerns about contagion on negative affect during the COVID-19 lockdown in adults: The moderating effect of gender and resilience. J Health Psychol. 2022; 27:1165–75. doi: 10.1177/1359105321990794 33541155 PMC8685745

[48] Zhang Q, Dong G, Meng W, Chen Z, Cao Y, Zhang M. Perceived Stress and Psychological Impact Among Healthcare Workers at a Tertiaty Hospital in China During the COVID-19 Outbreak: The Moderating Role of Resilience and Social Support. Front Psychiatry. 2022; 12:570971. doi: 10.3389/fpsyt.2021.570971 35281206 PMC8904916

[49] Daly M, Sutin AR, Robinson E. Longitudinal changes in mental health and the COVID-19 pandemic: evidence from the UK Household Longitudinal Study. Psychol Med. 2020: 1–10. doi: 10.1017/S0033291720004432 33183370 PMC7737138

[50] Robinson E, Sutin AR, Daly M, Jones A. A systematic review and meta-analysis of longitudinal cohort studies comparing mental health before versus during the COVID-19 pandemic in 2020. J Affect Disord. 2022; 296:567–76. doi: 10.1016/j.jad.2021.09.098 34600966 PMC8578001

[51] Morales-Vives F, Dueñas JM, Vigil-Colet A, Camarero-Figuerola M. Psychological Variables Related to Adaptation to the COVID-19 Lockdown in Spain. Front Psychol. 2020; 11: 565634. doi: 10.3389/fpsyg.2020.565634 33041929 PMC7525044

[52] Song S, Yang X, Yang H, Zhou P, Ma H, Teng C, et al. Psychological Resilience as a Protective Factor for Depression and Anxiety Among the Public During the Outbreak of COVID-19. Front Psychol. 2020; 11:618509. doi: 10.3389/fpsyg.2020.618509 33551929 PMC7862326

[53] Vannini P, Gagliardi GP, Kuppe M, Dossett ML, Donovan NJ, Gatchel JR, et al. Stress, resilience, and coping strategies in a sample of community-dwelling older adults during COVID-19. J Psychiatr Res. 2021; 138: 176–185. doi: 10.1016/j.jpsychires.2021.03.050 33862301 PMC8369528

[54] Li F, Luo S, Mu W, Li Y, Ye L, Zheng X, et al. Effects of sources of social support and resilience on the mental health of different age groups during the COVID-19 pandemic. BMC Psychiatry. 2021; 21: 16. doi: 10.1186/s12888-020-03012-1 33413238 PMC7789076

[55] Huang Y, Su X, Si M, Xiao W, Wang H, Wang W, et al. The impacts of coping style and perceived social support on the mental health of undergraduate students during the early phases of the COVID-19 pandemic in China: a multicenter survey. BMC Psychiatry. 2021; 21:530. doi: 10.1186/s12888-021-03546-y 34706690 PMC8549419

[56] Kaslow NJ, Friis-Healy EA, Cattie JE, Cook SC, Crowell AL, Cullum KA, et al. Flattening the emotional distress curve: A behavioral health pandemic response strategy for COVID-19. Am Psychol. 2020; 75:875–86. doi: 10.1037/amp0000694 32538638

[57] Heath C, Sommerfield A, von Ungern-Sternberg BS. Resilience strategies to manage psychological distress among healthcare workers during the COVID-19 pandemic: a narrative review. Anaesthesia. 2020; 75:1364–71. doi: 10.1111/anae.15180 32534465 PMC7323405

[58] Aiello A, Khayeri MY, Raja S, Peladeau N, Romano D, Leszcz M, et al. Resilience training for hospital workers in anticipation of an influenza pandemic. J Contin Educ Health Prof. 2011; 31:15–20. doi: 10.1002/chp.20096 21425355

[59] Maunder RG, Lancee WJ, Mae R, Vincent L, Peladeau N, Beduz MA, et al. Computer-assisted resilience training to prepare healthcare workers for pandemic influenza: a randomized trial of the optimal dose of training. BMC Health Serv Res. 2010; 10:72. doi: 10.1186/1472-6963-10-72 20307302 PMC2851711

[60] Panourgia C, Wezyk A, Ventouris A, Comoretto A, Taylor Z, Yankouskaya A. Individual factors in the relationship between stress and resilience in mental health psychology practitioners during the COVID-19 pandemic. J Health Psychol. 2021: 13591053211059393. doi: 10.1177/13591053211059393 34875921 PMC9483698

[61] Brooks SK, Webster RK, Smith LE, Woodland L, Wessely S, Greenberg N, et al. The psychological impact of quarantine and how to reduce it: rapid review of the evidence. Lancet. 2020 Mar 14;395(10227):912–920. doi: 10.1016/S0140-6736(20)30460-8 . 32112714 PMC7158942

[62] TMGH-Global COVID-19 Collaborative. Perceived Stress of Quarantine and Isolation During COVID-19 Pandemic: A Global Survey. Front Psychiatry. 2021 May 25;12:656664. doi: 10.3389/fpsyt.2021.656664 . 34113270 PMC8186534

